# Targeting and cytotoxicity of chimeric antigen receptor T cells grafted with PD1 extramembrane domain

**DOI:** 10.1186/s40164-023-00438-7

**Published:** 2023-09-30

**Authors:** Ang Zhang, Shenyu Wang, Yao Sun, Yikun Zhang, Long Zhao, Yang Yang, Yijian Zhang, Lei Xu, Yangyang Lei, Jie Du, Hu Chen, Lian Duan, Mingyi He, Lintao Shi, Lei Liu, Quanjun Wang, Liangding Hu, Bin Zhang

**Affiliations:** 1 Academy of Military Medical Sciences, Academy of Military Sciences, Beijing, 100850 PR China; 2 Senior Department of Hematology, the Fifth Medical Center of Chinese PLA General Hospital, Beijing, 100071 PR China; 3 Department of Hematology, Strategic Support Force Medical Center, Beijing, China; 4 SAFE Pharmaceutical Research Institute Co., Ltd, Beijing, China

**Keywords:** Chimeric antigen receptor T cell, PD1, Off-target toxicity, Hinge domain, Tumor microenvironment

## Abstract

**Background:**

Immunosuppression induced by programmed cell death protein 1 (PD1) presents a significant constraint on the effectiveness of chimeric antigen receptor (CAR)-T therapy. The potential of combining PD1/PDL1 (Programmed cell death 1 ligand 1) axis blockade with CAR-T cell therapy is promising. However, developing a highly efficient and minimally toxic approach requires further exploration. Our attempt to devise a novel CAR structure capable of recognizing both tumor antigens and PDL1 encountered challenges since direct targeting of PDL1 resulted in systemic adverse effects.

**Methods:**

In this research, we innovatively engineered novel CARs by grafting the PD1 domain into a conventional second-generation (2G) CAR specifically targeting CD19. These CARs exist in two distinct forms: one with PD1 extramembrane domain (EMD) directly linked to a transmembrane domain (TMD), referred to as PE CAR, and the other with PD1 EMD connected to a TMD via a CD8 hinge domain (HD), known as PE8HT CAR. To evaluate their efficacy, we conducted comprehensive assessments of their cytotoxicity, cytokine release, and potential off-target effects both in vitro and in vivo using tumor models that overexpress CD19/PDL1.

**Results:**

The findings of our study indicate that PE CAR demonstrates enhanced cytotoxicity and reduced cytokine release specifically towards CD19 + PDL1 + tumor cells, without off-target effects to CD19-PDL1 + tumor cells, in contrast to 2G CAR-T cells. Additionally, PE CAR showed ameliorative differentiation, exhaustion, and apoptosis phenotypes as assessed by flow cytometry, RNA-sequencing, and metabolic parameter analysis, after encountering CD19 + PDL1 + tumor cells.

**Conclusion:**

Our results revealed that CAR grafted with PD1 exhibits enhanced antitumor activity with lower cytokine release and no PD1-related off-target toxicity in tumor models that overexpress CD19 and PDL1. These findings suggest that our CAR design holds the potential for effectively addressing the PD1 signal.

**Supplementary Information:**

The online version contains supplementary material available at 10.1186/s40164-023-00438-7.

## Introduction

Chimeric antigen receptor (CAR)-T cell therapy has emerged as a groundbreaking approach in cancer treatment [1, 2]. While CAR-T has shown remarkable success in B-cell and plasma cell malignancies, it still faces significant challenges in solid tumors and hematological malignancies, particularly due to the immunosuppressive microenvironment [3–6]. Programmed cell death ligand 1 (PDL1) is frequently expressed in various solid tumors and hematologic malignancies, and its presence can hinder antitumor immunity [7–10]. The development of CAR-T cell exhaustion has been proposed to be triggered by co-inhibitory pathways such as PD1/PDL1 [11]. Consequently, researchers have explored combining CAR-T with PD1 blockade as a potential tool in immunotherapy [12]. However, the systemic use of PD1/PDL1 monoclonal antibodies can lead to immune-related adverse events (irAEs) owing to the crucial role of the PD1 pathway in maintaining immunologic homeostasis [13]. Several novel strategies have been developed to counteract PD1/PDL1 signaling in cancer treatment to mitigate the toxicity associated with systemic PD1 antibody use. These strategies include PD1-CD28 switch-receptor [14–17], PD1/PDL1 antibody single-chain variable fragment (scFv) secretion [18, 19], and PD1 knockdown or knockout [20, 21]. Despite their promising results, these approaches have limitations that must be addressed for clinical translation. For instance, PD1-CD28 CAR-T cells secrete higher levels of immune-activated cytokines, which increases the risk of cytokine release syndrome (CRS) [14]. Additionally, PD1 knockdown or knockout approaches may impair proliferative activity and promote T-cell exhaustion, potentially compromising the antitumor efficacy of CAR-T cells [18, 19, 22]. Therefore, it remains an open topic of exploration to determine how to effectively target tumor antigens with CAR-T while overcoming PD1 signaling from a novel perspective.

Some studies have demonstrated the cytotoxicity of CAR-T cells targeting PDL1 on tumor cells in preclinical studies; however, their clinical translation is hindered by safety concerns [23, 24]. Directly targeting PDL1 using CAR-T cells can lead to severe and systemic toxicity because of the widespread expression of PDL1 on hematopoietic and parenchymal cells [25]. The hinge region (HD) of the CAR provides the necessary flexibility and length to access the target antigen while overcoming steric hindrance [26, 27]. In our previous study, we demonstrated that reducing the flexibility of the hinge region can weaken the antigen-mediated activation of CAR-T cells [28]. Based on this, we hypothesize that the antigen-mediated activation of CAR for PDL1 can be modulated by deleting the HD, rendering PDL1 recognition insufficient to activate T cells. Therefore, we attempted to design a safe CAR that targets both a tumor antigen (such as CD19) and PDL1, with PDL1 playing a complementary role without directly killing only PDL1 + cells. This approach has the potential to overcome PD1-induced immunosuppression while avoiding PDL1/2-targeted toxicity.

Here we present a novel CAR design to effectively target and eliminate CD19+/PDL1 + tumor cells while avoiding PD1-related off-target toxicity. To restrain the PD1 signal, we incorporated PD1 extramembrane domain (EMD) into a conventional second-generation CAR (2G) structure. Two forms of this modified CAR were developed: one with PD1 EMD directly connected to a transmembrane domain (TMD), referred to as PE CAR, and another with PD1 EMD connected via a hinge domain (HD), known as PE8HT CAR. Additionally, two variations of the PE CAR were designed for validation, using either PD1 or CD8a TMD, named PEPT CAR and PE8T CAR, respectively. Our results demonstrate that both PE CAR variants exhibit superior cytotoxicity and reduced cytokine release when targeting CD19 + PDL1 + tumor cells, as compared to 2G CAR-T cells, both in vitro and in vivo. Importantly, no off-target effects were observed when targeting CD19-PDL1 + tumor cells in vitro. These findings highlight the potential of our novel CAR design to effectively target tumor antigens while overcoming PD1 signaling, thereby holding significant promise for future clinical applications.

## Methods

### Cell lines and cell culture conditions

Cell lines were cultured according to the manufacturers’ recommendations. NALM-6 is a pre-B cell acute lymphoblastic leukemia (ALL) cell line with high expression of CD19 (German DSMZ cell collection Cat#: ACC128). NALM-6-PDL1-GFP-luciferase (luc) is a stable cell line engineered to express PDL1-GFP-luc. K562 is a chronic myelogenous leukemia cell line (ATCC; Cat#: CCL-243). K562-CD19, K562-PDL1-GFP-luc, and K562-CD19-PDL1-GFP-luc are stable cell lines engineered to express CD19 and PDL1-GFP-luc. 786o is a renal cell adenocarcinoma cell line (ATCC; Cat#: CRL-1932™) that naturally expresses PDL1. CD19 was transduced into 786o using a lentivirus system to produce 786o-CD19. The aforementioned tumor cells were cultured in RPMI 1640 (Gibco, USA) supplemented with 10% heat-inactivated fetal calf serum (FCS) 100 U/mL penicillin, 100 mg/mL streptomycin sulfate, and 1% L-glutamine. T cells were cultured in X-VIVO15 (Lonza, USA) supplemented with 100 U/mL penicillin, 100 U/mL streptomycin sulfate, 1% L-glutamine, and 200 U/mL IL-2. The expression of CD19 and PDL1 of tumor cells is shown in sFig.1.

### Generation of CAR constructs

The conventional second-generation CAR (2G) structure in this study was constructed by fusing CD19 scFv, CD8α hinge and TMD, 4-1BB co-stimulatory domain, and CD3ζ signaling domain. The CAR with a PD1CD28 switch-receptor (PD1-S28) structure was created by fusing the second CAR with the “PD1CD28” chimeric receptor. The PE8HT CAR-T structure was formed by fusing CD19 scFv, a linker (G4S)4, a truncated extracellular PD1 (AA21-155), CD8 hinge and TMD, 4-1BB co-stimulatory domain, and CD3ζ signaling domain. The PEPT CAR-T structure was generated by fusing CD19 scFv, a linker (G4S)4, a truncated extracellular and transmembrane PD1 (AA21-191) derived from PD1 cDNA, 4-1BB co-stimulatory domain, and CD3ζ signaling domain. Finally, the PE8T structure was created by fusing CD19 scFv, a linker (G4S)4, a truncated extracellular PD1 (AA21-155) derived from PD1 cDNA, CD8 TMD, 4-1BB co-stimulatory domain, and CD3ζ signaling domain. These CAR structures were subcloned into viral vectors for transfection into activated T cells.

### Production of lentivirus particles

Lentiviruses were produced by transiently transfecting three plasmids into 293T cells using Lipo2000 (Invitrogen, USA). Briefly, 80% confluent 293T cells in 15-cm plates (Nalgene Nunc, USA) were transfected with approximately 30 µg of three plasmids, comprising 5 µg of the structural plasmid pHDH-Hgpm2 (HIV gag-pol), pMD-tat, pRC/CMV-rev, and Env VSV-G, and 10 µg of the vector encoding plasmid. Following a previously published protocol, the virus supernatant was concentrated using ultracentrifugation (Backman, USA) [29]. The concentrated virus was stored at − 80 °C.

### Selection, activation, and lentivector transduction of CD3 + T cells

Blood samples from healthy volunteers were obtained using an approved protocol by the Fifth Medical Center Ethics Committee of Chinese PLA General Hospital (Ethical code: Ky-2018-5-37), following the Declaration of Helsinki. All participants provided written informed consent before participating in the study. Human peripheral blood mononuclear cells (PBMCs) were separated using Ficoll-Paque PLUS. T cells were isolated using positive selection and stimulated with CD3/CD28 Dynabeads. After 48 h, activated T cells were transduced with lentivirus at a MOI of 5–10 and cultured in X-VIVO15 with 100 U/mL penicillin, 100 U/mL streptomycin sulfate, 1% L-glutamine, and 200 U/mL IL-2. The transduction efficiency for CAR-positive cells was determined by flow cytometry using a biotinylated human CD19 protein (Acrobiosystems, Cat. No. CD9-HF251, USA).

### Binding assay

To evaluate the function of PE-CARs and understand the underlying mechanisms, we used 2G and PD1-S28 CARs as controls [14]. To determine the affinity of different CAR-T cells for CD19 protein, we measured the fluorescence intensity of CAR-T cells at various concentrations of CD19 protein. The number of CAR-positive and mock-T cells was consistent with that of 2G CAR-T cells. Specifically, mock-T, 2G, and PEPT CAR-T cells were washed twice with PBS (1% BSA) by centrifugation. They were then treated with CD19-Fc protein (11,880-H02H) at different final concentrations (ranging from 180 µg/mL to 0.05 µg/mL). The cells were incubated at 4 °C in darkness for 45 min and washed twice with a PBS washing solution by centrifugation. Next, the cells were treated with 10 µL of goat anti-human IgG (FC)/FITC, incubated at 4 °C in darkness for 20 min, washed twice with a washing solution by centrifugation, and analyzed using flow cytometry (NovoCyte D3010).

### Cell proliferation

T cells were washed and then resuspended in 100 mL of PBS at a concentration of up to 1 × 10^7^ cells per mL. They were stained with 100 mL of 2.5 mM carboxyfluorescein diacetate succinimidyl ester (CFSE) (BioLegend, USA) to achieve a final concentration of 1.0 mM. This staining was performed for 20 min at 37 °C. The reaction was stopped by adding RPMI-1640 medium supplemented with 10% FBS, and the cells were washed twice. For the coculture assay, T cells were incubated with target cells at an effector-to-target (E:T) ratio of 1:1 for 48 h. The E:T ratio represents the ratio of the absolute number of CAR-T cells to target cells. The number of T cells used in the assay was the same as that of the 2G CAR-T group.

### Cytotoxicity assay

In the experiment, CFSE-labeled target cells were cocultured with effector T cells at the specified ratios for either 12–16 h or 6–8 h. After the coculture period, the cells were harvested and Annexin V and 7-AAD were added for flow cytometric analysis. The remaining live target cells were identified as CFSE + Annexin V- 7-AAD-. The E:T ratios represented the ratios of the absolute number of CAR-T cells to target cells. The number of mock-T cells used in the experiment was the same as that in the 2G CAR-T group. All experiments were conducted in triplicate to ensure reliability and consistency. To calculate the cytotoxicity efficiency of CAR-T cells, the ratio of dead target cells to the total number of dead and live cells was determined. Dead cells were identified as Annexin V + 7-AAD- or Annexin V + 7-AAD + cells.

### Cytokines production

To assess cytokine production, effector cells (5 × 10^4^) and target cells (5 × 10^4^) were cocultured at a 1:1 ratio in RPMI medium supplemented with 10% FBS and 10% human serum for 24 h. The concentration of cytokines in the culture supernatant and mouse serum was measured using enzyme-linked immunosorbent assay (ELISA) kits. Specifically, ELISA kits from MultiSciences Biotech Co., Ltd. (China) were used to measure the levels of IFN-γ, TNF-α, and IL-2. Additionally, a human premixed multi-analyte flow assay kit from BioLegend (USA) was used to simultaneously measure the concentration of a panel of soluble factors (IL-2, IL-6, IL-10, IL-21, IFN-γ, and TNF-α) in the same sample. The E:T ratio represented the ratio of the absolute number of CAR-T cells to target cells. The number of T cells used in the experiment was the same as that in the 2G CAR-T group.

### Flow cytometry

Anti-human antibodies were obtained from Becton Dickinson, BioLegend, and Miltenyi Biotec. The Accuri C6, FACS Calibur, and BD FACSAria™ II cell sorter were used for analyzing various samples. Anti-human antibodies were purchased from BioLegend, eBioscience, Acrobiosystems, or BD. Cells were isolated from in vitro cultures or animals, washed with PBS supplemented with 2% FCS, and stained on ice after blocking Fc receptors. In all analyses, the population of interest was gated based on forward vs. side scatter characteristics, followed by singlet gating. The differentiation stage of T cells was labeled as naïve T (T_N_): CD45RA + CD45RO-, stem cell memory T (T_SCM_): CD45RA + CD45RO+, central memory T (T_CM_): CD45RA-CCR7+, and effector memory T (T_EM_): CD45RA-CCR7- [30].

### Mouse xenograft tumor model

In the mouse xenograft tumor model, the animal experiments were conducted at the National Beijing Center for Drug Safety Evaluation and Research and at the SAFE Pharmaceutical Research Institute Co., Ltd. The study was conducted following the guidelines and regulations set by the Institutional Animal Care and Use Committee (IACUC-2019-001). Female NSG mice, aged 6–8 weeks, were used for the experiments. To establish the NALM-6-PDL1 acute precursor B-ALL models, 10^6^ tumor cells were intravenously injected with PBS. Tumor growth was monitored by measuring the total bioluminescent flux using a Xenogen Imaging System (PerkinElmer-IVIS Lumina III). Peripheral blood samples were collected from the mice via the tail vein. Furthermore, different organs were dissected from the NSG mice, and cells from these organs were harvested. The expression of murine PDL1/2 in different tissues was detected. Due to the cross-species binding affinity of human PD1 to murine PDL1/2, human PDL1/2 antibodies were used to detect the expression of murine PDL1/2.

### RNA-sequencing and bioinformatics analysis

Total RNA was purified from 2G CAR-T cells or PEPT/PE8T CAR-T cells after incubating with NALM-6 cells with a RNeasy Mini Kit according to the manufacturer’s instructions (Qiagen). RNA integrity was verified with an Agilent TapeStation (RIN). The further preparation for sequencing and bioinformatics analysis was performed as previously described [31].

### Analysis of metabolic parameters

Basal oxygen consumption rate (OCR) was measured, followed by serial additions of oligomycin (an inhibitor of ATP synthesis), carbonyl cyanide-ptrifluoromethoxyphenylhydrazone (FCCP; an uncoupling ionophore), and rotenone with antimycin A (blocking agents for complexes I and III of the electron transport chain, respectively) to discern the relative contributions of mitochondrial and non-mitochondrial mechanism of oxygen consumption [49]. Mitochondrial function was assessed with an extracellular flux analyzer (Seahorse Bioscience). Individual wells of an XF96 cell culture microplates were coated with CellTak in accordance with the manufacturer’s instructions. The matrix was adsorbed overnight at 37 °C, aspirated, air-dried, and stored at 4 °C until use. Mitochondrial function was assessed on day 10 (incubated with NALM-6-PDL1 for 48 h). To assay mitochondrial function, we centrifuged T cells at 500 × *g* for 5 min. Cell pellets were resuspended in XF assay medium (non-buffered RPMI 1640) containing 5.5 mM glucose, 2 mM L-glutamine, and 1 mM sodium pyruvate and seeded at 5 × 10^4^ cells per well. The microplate was centrifuged at 500 × *g* for 5 min and incubated in standard culture conditions for 60 min. During instrument calibration (30 min) the cells were switched to a CO_2_-free (37 °C) incubator. XF96 assay cartridges were calibrated in accordance with the manufacturer’s instructions. Cellular OCRs were measured under basal conditions and, following treatment with 1.5 mM oligomycin, 1.5 mM FCCP, and 40 nM rotenone, with 1mM antimycin A (XF Cell Mito Stress kit, Seahorse Bioscience).

### Statistical analysis

Statistical analyses were performed using Prism version 7.0 (GraphPad). For studies comparing two groups, we utilized an unpair Students *t*-test. Survival data were analyzed by the log-rank test, and survival curves were assessed using the Kaplan-Meier method.

## Results

### CAR grafted with PD1, without the use of a HD, exhibited enhanced cytotoxicity towards CD19 + PDL1 + tumor cells, without causing PD1-related-off-target toxicity

**Fig. 1:**
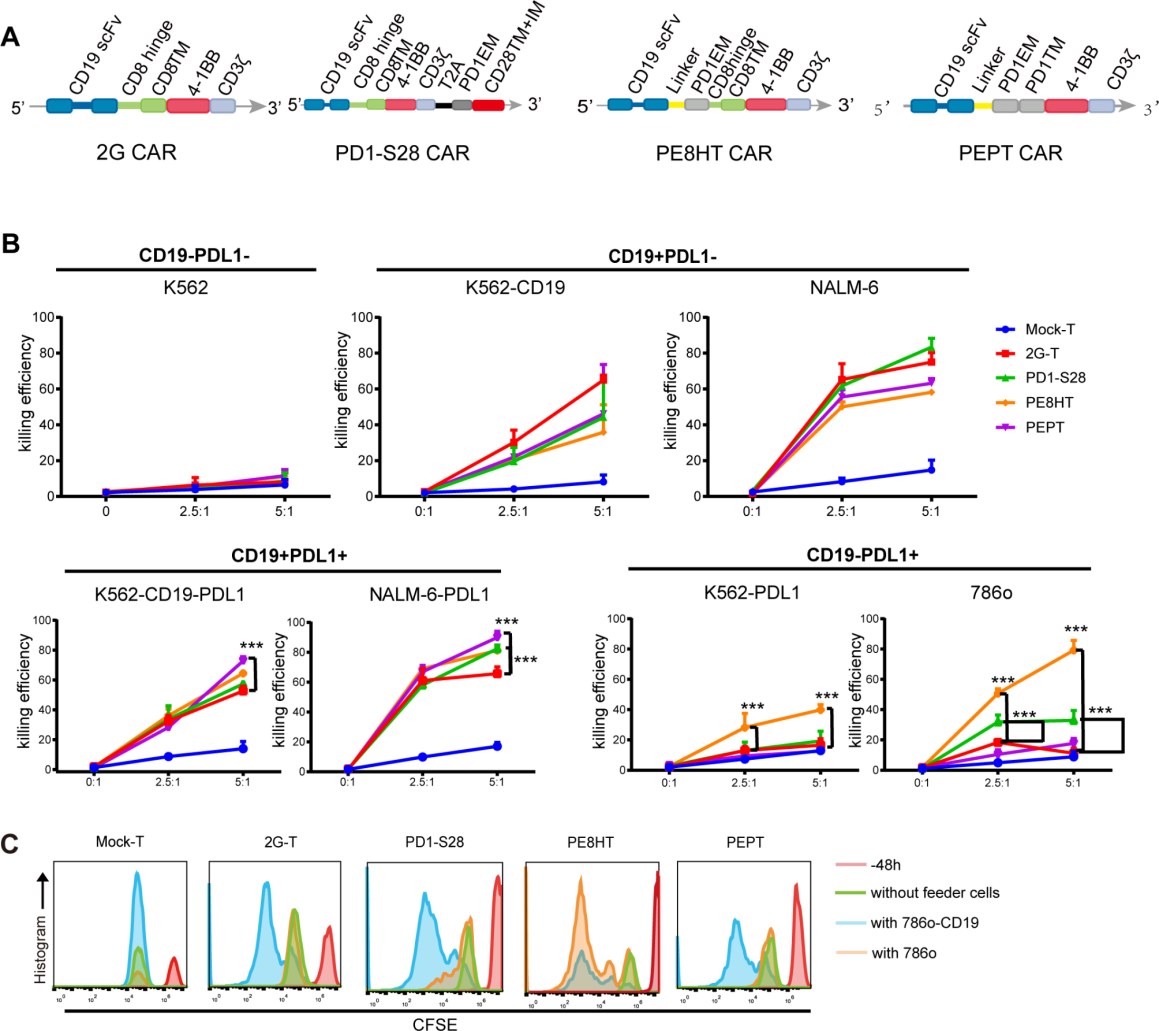
CAR grafted with PD1 with or without CD8 hinge exhibited different function to PDL1. **A.** Schematic representation of four CAR variants with different domains: 2G, PD1-S28, PE8HT, and PEPT CAR, showing variations in the hinge, extramembrane, and transmembrane domains. **B.** Cytotoxic percentages of targeted cells by mock-T and 2G, PD1-S28, PE8HT, and PEPT CAR-T cells were measured after 12–16 h of coculture in vitro. The E:T ratios (2.5:1 and 5:1) represent the ratios of the absolute number of CAR-T cells to target cells. The following cell types were used: CD19-PDL1- cells (K562), CD19 + PDL1- cells (K562-CD19 and NALM-6), CD19 + PDL1 + cells (K562-CD19-PDL1 and NALM-6-PDL1), and CD19-PDL1 + cells (K562-PDL1 and 786o). The number of mock-T cells was the same as in the 2G group. The results shown are representative of at least three independent experiments using T cells from different healthy donors. **C.** Proliferation of different effector cells was assessed after stimulation with CD19-PDL1+ (786o) and CD19 + PDL1+ (786o-CD19) cell lines. All effector cells were stained with CFSE. Proliferation was analyzed by flow cytometry by measuring CFSE dilution. The red, green, orange, and blue peaks represent cells before stimulation, cells without any additional stimulation after 48 h, cells incubated with 786o cells (at a 1:1 ratio of 10^5^:10^5^) after 48 h, and cells incubated with 786o-CD19 cells (at a 1:1 ratio of 10^5^:10^5^) after 48 h. CAR: chimeric antigen receptor; CFSE: carboxyfluorescein diacetate succinimidyl ester

Two different constructs of the CAR, namely PE8HT CAR and PEPT CAR, were designed. The PE8HT CAR included domains of linker, PD1 EMD, CD8 Hinge, and TMD between CD19scFv and 41BB, while the PEPT CAR included domains of linker, PD1 EMD, and TMD. The schematic diagram of these different CAR constructs used in our study is shown in Fig. 1A.

When challenged with CD19 + PDL1- target cells (NALM-6 and K562-CD19), all anti-CD19 CAR-T cells exhibited similar cytotoxicity compared to 2G CAR-T cells and did not induce killing of CD19-PDL1- target cells (K562) (Fig. 1B). However, when challenged with CD19 + PDL1 + target cells (NALM-6-PDL1 and K562-CD19-PDL1), PD1-S28 CAR-T, PE8HT CAR-T, and PEPT CAR-T cells demonstrated superior lysis compared to 2G CAR-T cells at an E:T ratio of 5:1 (Fig. 1B). Interestingly, PE8HT CAR-T cells exhibited specific cytotoxicity when challenged with CD19-PDL1 + target cells (786o and K562-PDL1), whereas PEPT CAR-T cells did not show the same response (Fig. 1B).

Different CAR-T cells were cocultured with target cells (786o-CD19 and 786o) to assess the proliferation of CAR-T cells towards CD19 + PDL1 + or PDL1 + target cells. Stimulation with 786o-CD19 cells (CD19 + PDL1+) resulted in significantly higher proliferation rates for all CAR-T cells. Following stimulation with 786o cells (CD19-PDL1+), PE8HT CAR-T cells exhibited an extremely high proliferation rate. In contrast, the proliferation rate of PEPT CAR-T cells was similar to that of 2G CAR-T cells (Fig. 1C).

### PEPT CAR demonstrates reduced antigen-specific cytokine release

**Fig. 2:**
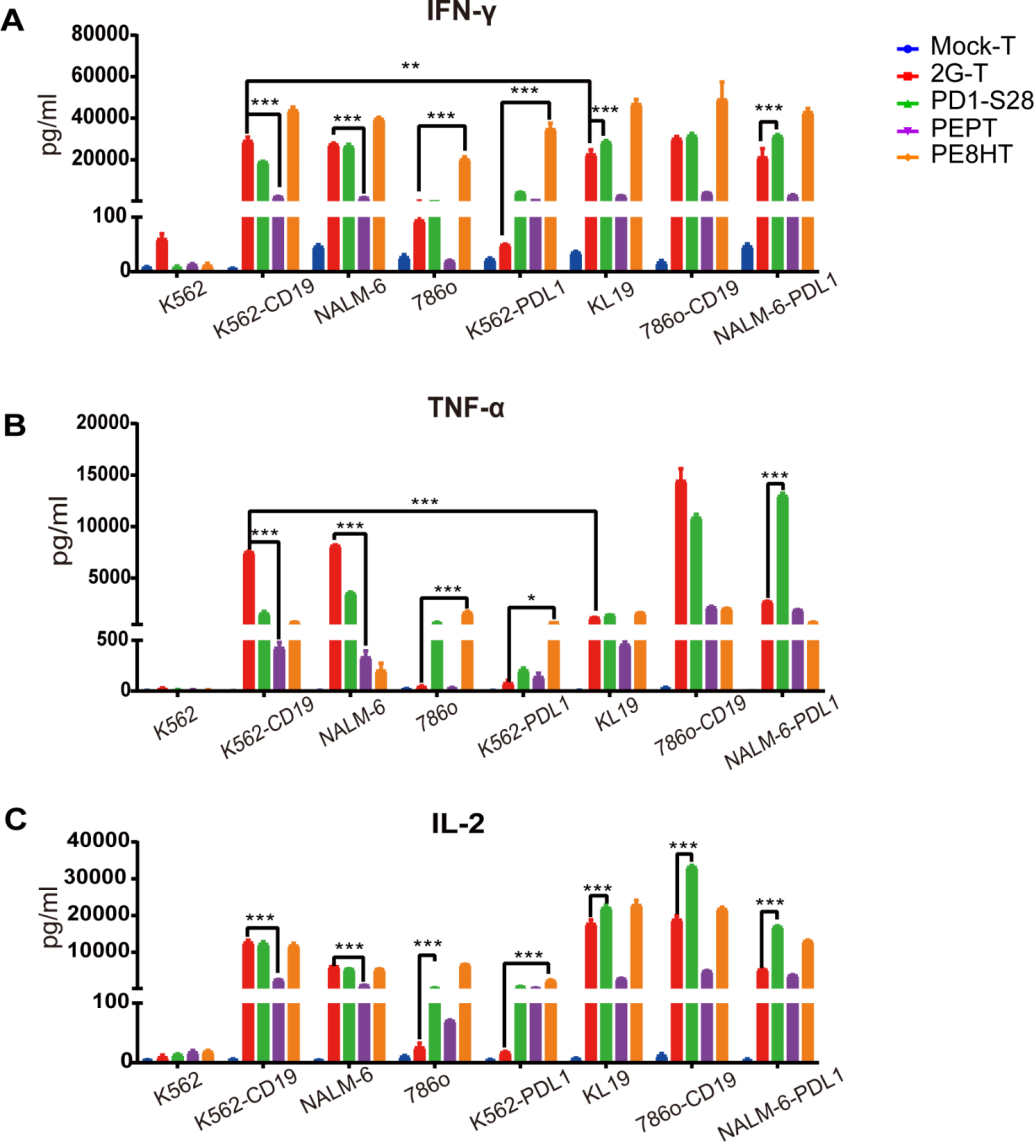
The proinflammatory cytokine release specific to the antigen was lower in PEPT CAR cells. **A–C**. IFN-γ, TNF-α, and IL-2 production by mock, 2G CAR, PD1-S28 CAR, PEPT CAR, and PE8HT CAR T cells. Cytokine concentrations in the media were measured after 24 h coincubation with K562, K562-CD19, NALM-6, 786o, K562-PDL1, K562-CD19-PDL1(KL19), 786o-CD19, and NALM-6-PDL1 cells at E:T of 1:1. Results are mean values ± SD of triplicate from one of two representative experiments. *P < 0.05; **P < 0.01; ***P < 0.005

To further evaluate the effector function of different CAR-T cells upon antigen-specific stimulation, we conducted a coculture assay to measure the release of cytokines (IFN-γ, TNF-α and IL-2) after incubation with tumor cells (Fig. 2). All CAR-T cells exhibited cytokine release in response to CD19 + tumor cells compared to mock T cells. However, only PEPT CAR-T cells displayed lower levels of IFN-γ, TNF-α, and IL-2 release compared to 2G CAR-T cells when challenged with CD19 + PDL1- tumor cells. Alternatively, both PD1-S28 and PE8HT CAR-T cells exhibited higher cytokine levels when exposed to CD19 + PDL1 + tumor cells. Specifically, PD1-S28 CAR-T cells displayed higher IL-2 release in KL19 (K562-CD19-PDL1), 786o-CD19, and NALM-6-PDL1, TNF-α released higher in NALM-6-PDL1, and IFN-γ released higher in KL19 and NALM-6-PDL1 compared to 2G CAR-T cells. PE8HT CAR-T cells exhibited higher IFN-γ and IL-2 release than 2G CAR-T cells in KL19, 786o-CD19, and NALM-6-PDL1 (Fig. 2A, C). Furthermore, PE8HT CAR-T cells demonstrated a significant release of cytokines when incubated with 786o and K562-PDL1 cells (CD19-PDL1+), indicating potential off-target effects owing to the expression of PDL1. When challenged with 786o cells, PD1-S28 CAR-T cells released higher levels of TNF-α and IL-2 compared to 2G CAR-T cells (Fig. 2B, C).

### PEPT CAR sustained antitumor activity in NALM-6-PDL1 bearing mice without PD1-related off-target toxicity

**Fig. 3:**
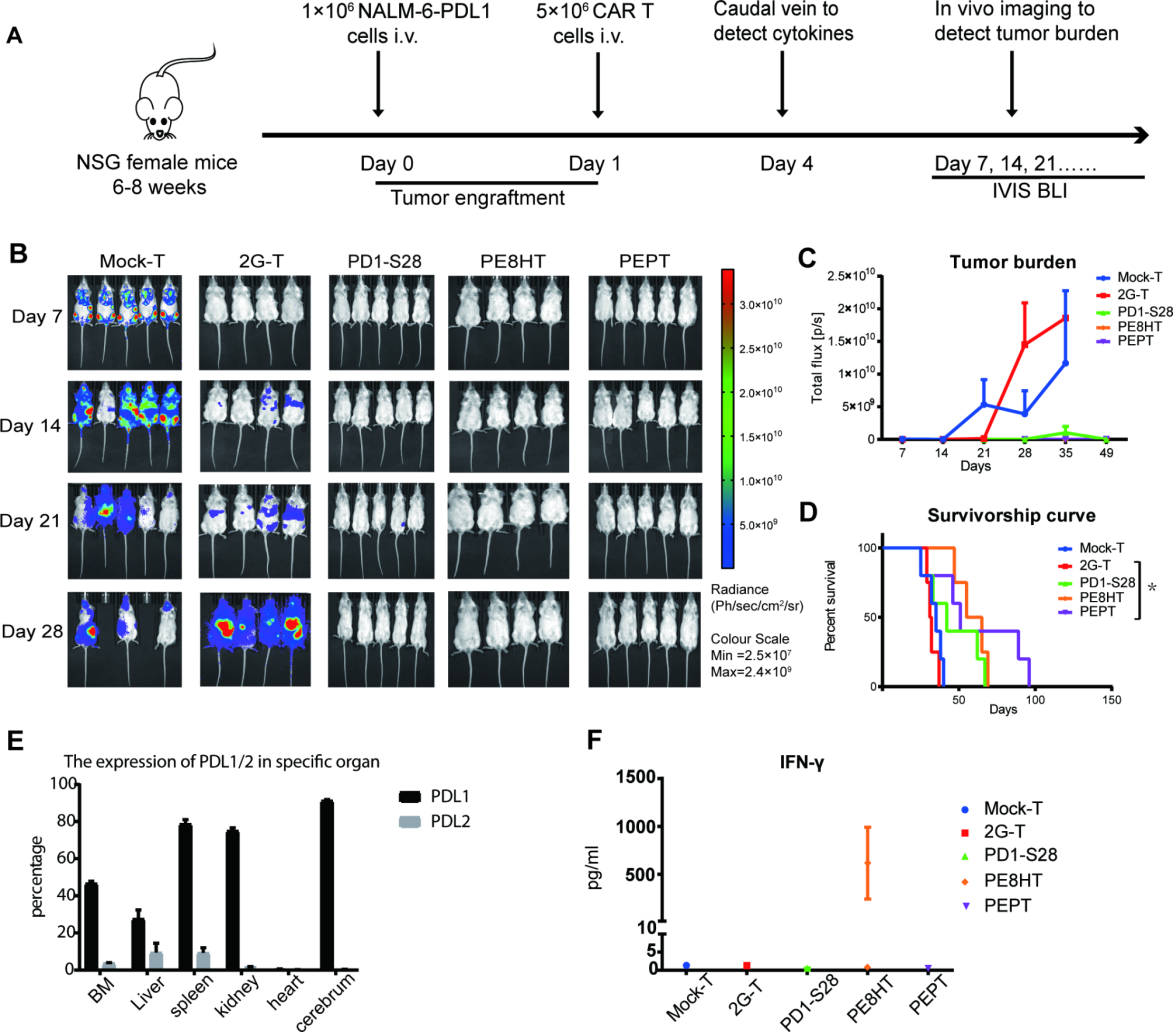
The PEPT CAR-T cells enhanced anti-tumor activity without off-target toxicity to PDL1 in vivo. **A**. Schematic representation of the experimental procedure for tumor challenge, T cell adoptive transfer, cytokine detection, and in vivo imaging. **B**. Representative bioluminescent images of tumor growth over time. **C**. Total body flux (photons/s) for each mouse was quantified and averaged per group. Error bars represent mean ± SEM.**D**. Kaplan-Meier survival analysis of NALM-6-PDL1-GFP-luc challenged mice. Overall survival curves were plotted using the Kaplan-Meier method and compared using the log-rank (Mantel-Cox) test (n = 4 or 5; * P < 0.05). **E**. Murine PDL1/2 expression in different tissues of NSG mice stained with human PDL1/2 antibodies by flow cytometry. **F**. On day 4, blood was collected from the tail vein. The samples from all mice of each group were pooled, and plasma was isolated. The concentration of IFN-γ was detected using an ELISA-kit in duplicate

We have observed that PEPT CAR-T cells exhibit stronger cytotoxicity against CD19 + PDL1 + tumors compared to 2G CAR-T cells in vitro. To further evaluate the antitumor efficacy of PEPT CAR-T cells, we used a xenograft mouse model implanted with CD19 + PDL1 + tumor cells (Fig. 3A). Initially, all CAR-T cell types demonstrated improved tumor control as observed through bioluminescence imaging compared to the mock-T cell group. However, the tumor burden in mice treated with 2G CAR-T cells rapidly relapsed after 2 weeks, failing to extend the survival of the animals (Fig. 3B-D). In contrast, while some mice treated with PD1-S28, PE8HT, or PEPT CAR-T cells succumbed to the tumor, others exhibited a low tumor burden after 7 weeks (Fig. 3C**)**. Notably, mice treated with PEPT CAR-T cells displayed significantly prolonged overall survival compared to those administered 2G CAR-T cells (Fig. 3D).

In contrast, mice treated with PE8HT CAR-T cells exhibited severe side effects at days 60 and 62, including depilation and irritability (**sFig. 2**). Additionally, we observed higher levels of PDL1 expression in multiple organs of these mice (Fig. 3E). Furthermore, only mice treated with PE8HT CAR-T cells experienced a significant increase in serum IFN-γ levels on day 3 (Fig. 3F).

### PE CAR with CD8 TMD demonstrates enhanced cytotoxicity and reduced cytokine release compared to 2G CAR-T cells in vitro and in vivo

**Fig. 4:**
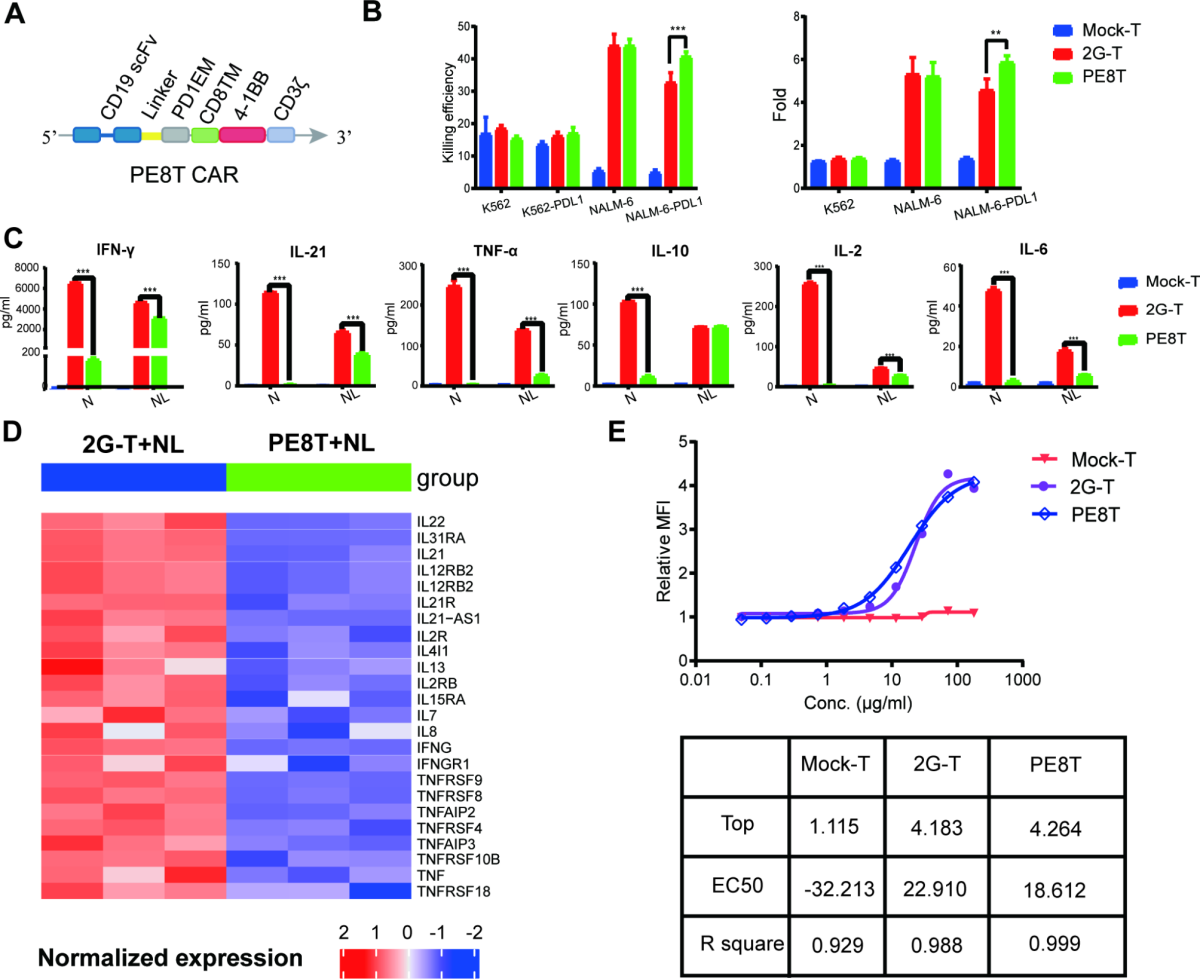
PE8T cells improved antitumor activity to CD19 + PDL1 + cells with reduced cytokine release and affinity for CD19 protein. **A.** Schematic of PE8T CAR containing variations in the hinge, extramembrane, and transmembrane domains. **B**. Cytotoxic percentages of K562, K562-PDL1, NALM-6, and NALM-6-PDL1 cells cocultured with mock-T, 2G, and PE8T CAR-T cells at E:T of 5:1 after 6–8 h in vitro. The results are representative of at least three independent experiments with T cells from different healthy donors (left); the proliferation of different effector cells after CFSE-staining, incubation with different target cells (K562, NALM-6, NALM-6-PDL1) in RPMI (10% FBS) for 48 h. The proliferation rate was analyzed by flow cytometry. (right) Results are mean values ± SD from three independent experiments. *P < 0.05; **P < 0.01; ***P < 0.005. **C**. IFN-γ, IL-21, TNF-α, IL-10, IL-2, and IL-6 production by mock-T, 2G, and PE8T cells. The concentration of cytokines in the media was measured after 24 h coincubation with N(NALM-6) and NL(NALM-6-PDL1) at E:T of 1:1 (mean ± SD, n = 2). Results are mean values ± SD of triplicate from one of two representative independent experiments. *P < 0.05; **P < 0.01; ***P < 0.005. **D**. Heat map of selected cytokines enriched in genes significantly upregulated or downregulated in 2G CAR-T vs. PE8T CAR-T cells after stimulation with NALM-6-PDL1 cells at E:T of 1:1 for 48 h. For each pathway, a single sample enrichment score was calculated, and the mean was taken per response group. A color gradient ranging from dark blue to dark red indicates the mean normalized enrichment score (ranging from − 2 to + 2) of pathways enriched in induced (red) or repressed (blue) genes. **E**. The affinity of CD19 protein to different CAR-T cells: 2G > PE8T. EC50 of CAR-T cells binding to CD19 protein was determined by flow cytometry. The results of D and E are mean values ± SD of triplicate from a single experiment. EC50, 50% maximal effective concentration

To further investigate the influence of the TMD, we designed and constructed a novel CAR called PE8T CAR by replacing the TMD of PD1 with CD8 TMD in the PEPT CAR construct (Fig. 4A).

Experiments were conducted to evaluate the effector function of PE8T CAR-T cells in vitro. When challenged with NALM-6-PDL1 cells, PE8T CAR-T cells exhibited stronger cytotoxicity and proliferation compared to 2G CAR-T cells, while no significant difference was observed when challenged with NALM-6 cells (Fig. 4B). Additionally, we measured the release of six cytokines (IFN-γ, IL-21, TNF-α, IL-10, IL-2, and IL-6) after incubating PE8T CAR-T cells with NALM-6 and NALM-6-PDL1 cells. PE8T CAR-T cells displayed moderate cytokine release in response to CD19 + tumor cells; however, not to CD19 − PDL1 + tumor cells, in contrast to 2G CAR-T cells (Fig. 4C). Gene set enrichment analysis (GSEA) revealed that PE8T CAR-T cells exhibited a decrease transcriptome level of proinflammatory cytokines compared to 2G CAR-T cells when stimulated with NALM-6-PDL1 cells (Fig. 4D). This finding was consistent with the results obtained from PEPT CAR-T cells (**sFig. 3**). An affinity test demonstrated that the 50% maximal effective concentration (EC50) of PE8T CAR-T cells for binding to CD19 protein was lower than that of 2G CAR-T cells, suggesting improved binding affinity (Fig. 4E). Furthermore, the expression efficiency of PE8T CAR was significantly improved, achieving approximately 60% compared to around 20% for PEPT CAR-T cells (**sFig. 4**).

**Fig. 5:**
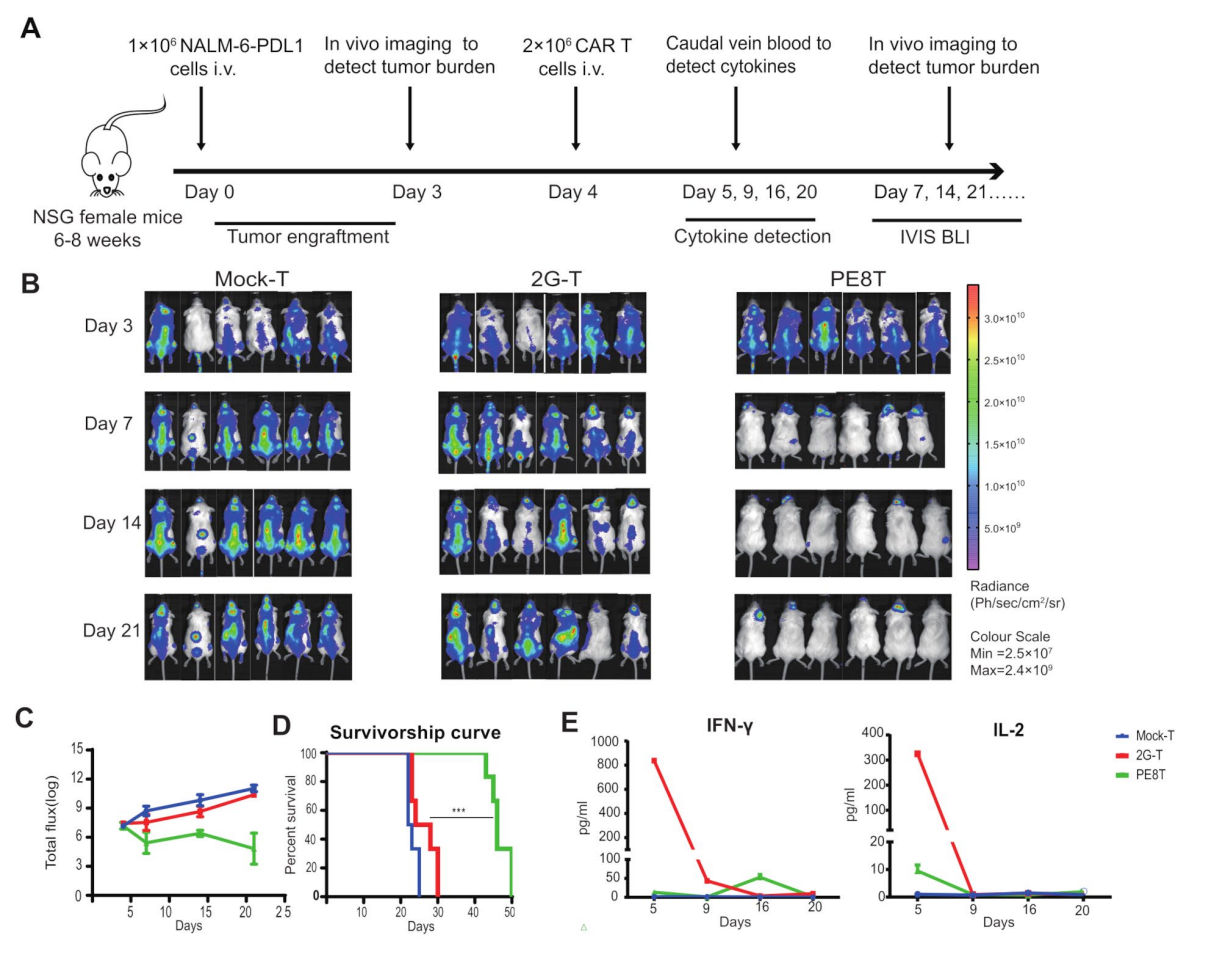
PE8T CAR-T cells harnessed tumor and prolonged survival in vivo compared with 2G CAR-T cells. **A.** Schematic representation of the experimental procedure for tumor challenge, T cell adoptive transfer, cytokines detection, and in vivo imaging. **B.** Representative bioluminescent images of tumor over time. **C.** The logarithm of total body flux (photons/s) for each mouse was quantified and averaged per group (mean ± SEM, n = 6). **D.** Kaplan–Meier survival analysis of NALM-6-PDL1-GFP-luc challenged mice. Overall survival curves were plotted using the Kaplan–Meier method and compared utilizing the log-rank (Mantel–Cox) test (n = 6). *P < 0.05; **P < 0.01; ***P < 0.005. **E**. On day 5, 9, 16, 20, blood was collected from one group mixed to detect the concentration of IFN-γ and IL-2 using an ELISA-kit (mean ± SD, n = 2)

Next, we evaluated the antitumor efficacy of PE8T CAR-T cells in a mouse model with established xenografts (Fig. 5A). PE8T CAR-T cells significantly reduced tumor burden and prolonged overall survival compared to 2G CAR-T cells (Fig. 5B-D). To verify cytokine release triggered by CAR-T cells in vivo, we measured IFN-γ and IL-2 levels in the serum after CAR-T cell infusion. Intense cytokine release was observed only in the 2G CAR-T cell group on day 5, while the level of cytokine release by PE8T CAR-T cells was comparable to that of the mock-T cell group (Fig. 5E).

### PE8T CAR-T cells exhibit improved phenotypes of exhaustion, differentiation, and apoptosis upon encountering CD19 + PDL1 + tumor cells

**Fig. 6:**
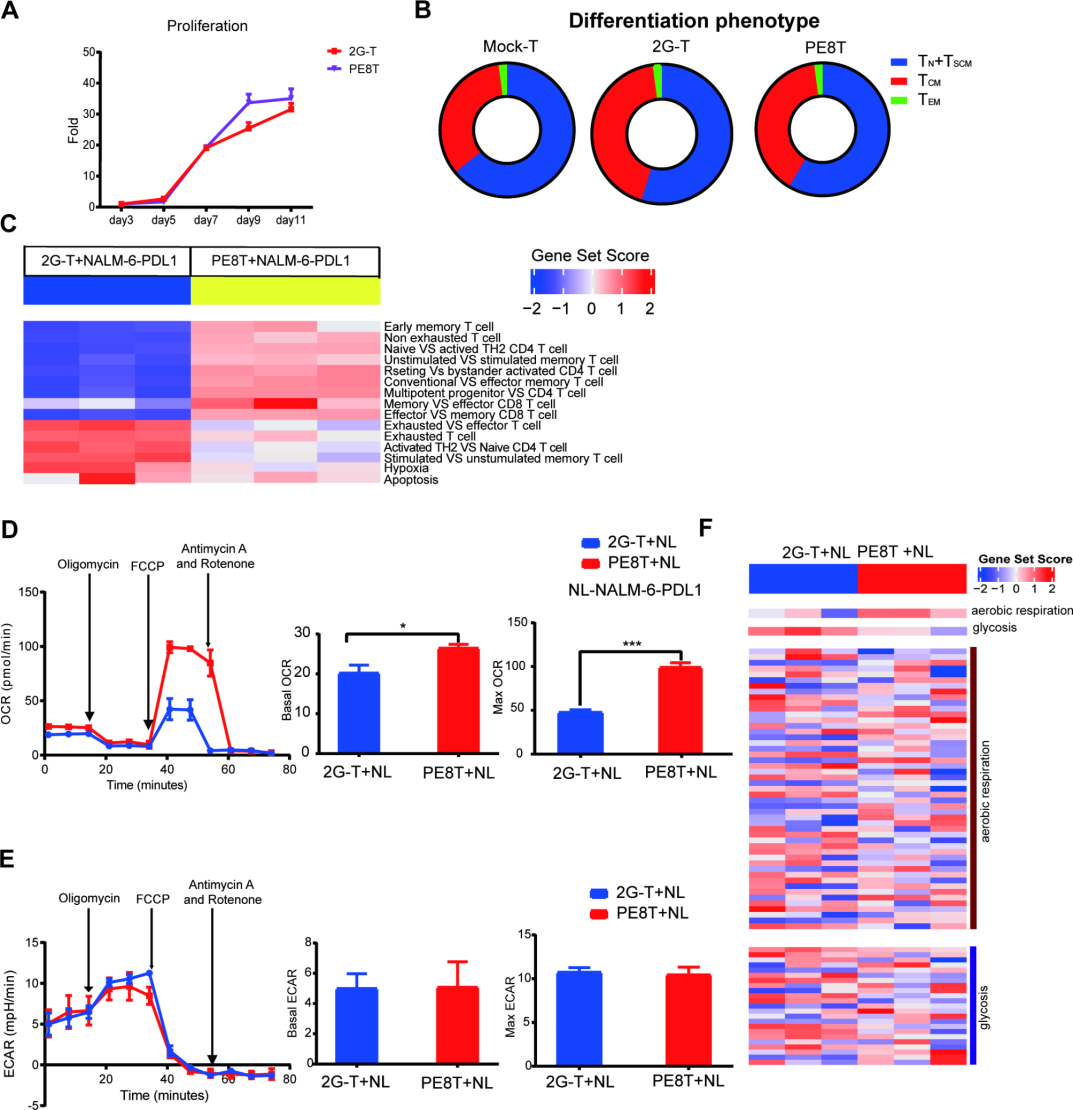
The PE8T cells reduced exhaustion and increased oxygen consumption rate inculcated with CD19 + PDL1 + tumor cells. **A**. Proliferation curves of different CAR-T cells from day 3 to day 11 after isolation (mean ± SD, n = 3). Results are representative of triplicate with T cells from one healthy donor. **B.** The stage of T cell differentiation and development is defined as: naïve T (T_N_): CD45RA + CD45RO−, stem cell memory T (T_SCM_): CD45RA + CD45RO+, central memory T (T_CM_): CD45RA + CCR7+, and effect memory T (T_EM_): CD45RA − CCR7−. Phenotypic detection was performed on day 8 after isolation (mean ± SD, n = 3). Results are representative of triplicate with T cells from one healthy donor. 2G CAR-T vs.PE8T CAR-T.**C**. Representative GSEA results from running the 2G CAR-T vs. PE8T CAR-T cells rank list about differentiation gene ontology sets (mean ± SD, n = 3). Results are representative of triplicate with T cells from one healthy donor. CAR-T cells were collected after stimulation with NALM-6-PDL1 cells at E:T of 1:1 for 48 h. **D**. The OCR, basal OCR and maximum OCR of 2G CAR-T and PE8T CAR-T cells in culture under basal metabolic conditions and in response to mitochondrial inhibitors, as specified in the experimental procedures. **E**. ECAR, basal ECAR and maximum ECAR of 2G CAR-T and PE8T CAR-T cells in culture under basal metabolic conditions and in response to mitochondrial inhibitors, as specified in the experimental procedures. Data represent at least triplicate performed with cells from one healthy human donor plotted as mean ± SD (*P < 0.05; **P < 0.01; ***P < 0.005.). One of three independent experiments is presented (n = 3). Data are represented as mean ± SD.**F.** Representative GSEA results from running the 2G CAR-T vs. PE8T CAR-T cells rank list about metabolism gene ontology sets (mean ± SD, n = 3). Results are representative of triplicate with T cells from one healthy donor. CAR-T cells were collected after stimulation with NALM-6-PDL1 cells at E:T of 1:1 for 48 h. One of three independent experiments is presented (n = 3). Data are represented as mean ± SD. ECAR, extracellular acidification rate; GSEA, gene set enrichment analysis; OCR, oxygen consumption rate

To better understand the fate of different engineered T cells, we characterized their phenotypes in vitro. PE8T CAR-T cells demonstrated a proliferation rate similar to that of 2G CAR-T cells (Fig. 6A). Upon stimulation with CD3/CD28 dynamic beads, a comparable fraction of PE8T CAR-T cells displayed stem cell memory T cell (T_SCM_) and central memory T cell (T_CM_) phenotypes compared to 2G CAR-T cells (Fig. 6B). To further investigate the differentiation phenotype after CAR-T cells encountered CD19 + PDL1 + tumor cells, we validated the reported gene signature associated with CAR-T cell efficacy through transcriptomic profiling [32]. PE8T CAR-T cells exhibited enrichment in memory-related genes and demonstrated lower levels of effector differentiation, exhaustion, and apoptosis compared to 2G CAR-T cells when challenged with CD19 + PDL1 + tumor cells (Fig. 6C and **sFig. 5**). Similar results were observed in PEPT CAR-T cells (**sFig. 6**). After coculturing with NALM-6 cells in vitro, both PE8T and PEPT CAR-T cells demonstrated a significant downregulation of PD1, PDL1, TIM-3, and CTLA-4 compared to 2G CAR-T cells (**sFig. 7**). In summary, PE8T and PEPT CAR-T cells exhibit improved phenotypes of exhaustion, differentiation, and apoptosis upon encountering CD19 + PDL1 + tumor cells.

### PE8T CAR-T cells exhibited an increased oxygen consumption rate when encountering CD19 + PDL1 + tumor cells

To investigate potential metabolic differences between 2G CAR-T and PE CAR-T cells, we measured the OCR and extracellular acidification rate (ECAR) as indicators of oxidative phosphorylation and glycolysis, respectively. Following stimulation with CD19 + PDL1 + tumor cells for 48 h, we observed an increase in basal OCR and a significant increase in maximal respiratory capacity in the PE8T CAR-T group compared to 2G CAR-T cells (maximal OCR: PE8T CAR-T > 2G CAR-T, ***; Fig. 6D). We also assessed the ECAR as a proxy for lactic acid production during glycolysis and found no significant difference between the PE8T CAR-T and 2G CAR-T groups (Fig. 6E). Additionally, we analyzed the transcriptome differences in metabolism among the different CAR-T cell groups after incubation with NALM-6-PDL1 cells for 48 h. Consistent with the in vitro findings, GSEA revealed that PE8T CAR-T cells were enriched in aerobic respiration-related genes and exhibited lower levels of glycolysis-related genes after stimulation with NALM-6-PDL1 cells (Fig. 6F).

## Discussion

In this study, we presented the findings demonstrating the superior cytotoxicity of PE CAR-T cells against CD19 + PDL1 + tumor cells, accompanied by a reduction in cytokine release both in vitro and in vivo. Notably, PE CAR-T cells did not exhibit toxicity towards CD19-PDL1 + cells in vitro. Our results suggest that the absence of HD in the CAR structure weakens the binding of extrinsic PD1 to its receptor, PDL1, thereby preventing T-cell activation in the presence of PDL1 alone. This low-avidity CAR targeting PDL1 contributes to our novel CAR design’s enhanced efficacy and safety. Furthermore, we found that PE8T CAR-T cells maintained comparable CAR expression efficacy, exhibited ameliorative phenotypes of differentiation, exhaustion, and apoptosis, and displayed increased OCR compared to 2G CAR-T cells in vitro. Additionally, we demonstrated that PE8T CAR with CD8a TMD improved CAR expression efficacy and retained similar functionality compared to PD1 TMD, making it more suitable for potential clinical applications. Overall, our study highlights the ability of PE CAR-T cells to overcome the immunosuppressive signal of the PD1/PDL1 axis and enhance potential efficacy compared to 2G CAR-T cells.

While the combination of dual-targeted CARs against CD19, CD20, CD22, and BCMA has shown promising clinical efficacy [33–36], directly targeting PDL1/2 with CAR-T cells raises safety concerns owing to the important role of PD1-PDL1/2 signaling in maintaining immune balance in normal tissues [37, 38]. Therefore, replacing one of the targets of the dual-targeted CAR with the EMD of PD1 is not a feasible solution, as it could lead to severe off-target effects. Our study demonstrated that PE8HT CAR, not PE CAR, exhibited a specific killing effect on CD19-PDL1 + cells. This suggests that the absence of HD reduces the ability of the CAR to recognize PDL1 and initiate activation signals. This finding aligns with our hypothesis that deleting the HD reduces the flexibility of the CAR, resulting in insufficient activation of T cells by PDL1 recognition alone. The improved function of these novel CAR-T cells may be attributed to their enhanced aerobic metabolism and reduced differentiation [39], as previously described [40, 41]. Further research is needed to fully understand the specific effect of HD deletion on CAR activation, particularly in the context of PDL1 recognition and immune synapse formation.

Nonsignaling elements within CAR constructs is crucial in the expression efficiency of CAR-T cells [42, 43]. CD8-mediated dimerization of the CAR enhances its transport from the endoplasmic reticulum to the cell surface, resulting in higher CAR expression levels [44]. CAR constructs incorporating the CD8 TMD have been shown to exhibit greater CAR expression compared to those incorporating the PD1 TMD, as PD1 functions as a monomer while CD8 can form dimers [45, 46]. Furthermore, studies have demonstrated that increasing the length of the hinge and transmembrane domains of the CAR can reduce cytokine release and promote higher expression of anti-apoptotic molecules [47]. Consistent with these findings, our study observed that CAR-T cells with PD1 TMD exhibited longer nonsignaling CAR domains than 2G CAR-T cells when targeting CD19; this may explain the decreased cytokine secretion observed when these CAR-T cells encountered target cells.

Previous studies have highlighted the importance of enhancing mitochondrial metabolic function and reducing cellular differentiation to improve the antitumor activity of CAR-T cells. Grafting CAR with PD1 EMD has been shown to promote CAR-T cells’ memory phenotype and aerobic metabolism of, potentially enhancing their persistence in the body [48]. Furthermore, Kawalekar et al. [49, 50] research demonstrated that incorporating the 4-1BB co-stimulatory domain into CAR-T cells can enhance mitochondrial metabolism and reduce glycolysis levels compared to using CD28 as a co-stimulatory molecule. This increase in mitochondrial mass can provide a survival advantage to CAR-T cells. Considering these findings, the increased oxygen consumption resulting from grafting PD1 EMD into CAR-T cells may contribute to their improved antitumor efficacy against NALM-6-PDL1.

This study acknowledges certain limitations that should be addressed in future research. Firstly, the efficacy and safety of PE8T in animal models bearing CD19 + PDL1- and CD19-PDL1 + cells were not further investigated, and a parallel comparison of in vitro and in vivo functions between the PE8T CAR T group and other groups was not conducted. These limitations weaken the overall quality of the study, and efforts will be made to improve upon them in future investigations. Notably, the persistent overexpression of PDL1 in tumor cells may have inhibited the specific antitumor activity of 2G CAR T cells against CD19 + PDL1 + tumor cells in vivo.

Additionally, the expression of PDL1 on T cells upon activation has been overlooked in many studies. PDL1 signaling on memory T cells is crucial in resolving inflammatory responses and maintaining a tolerogenic environment [51, 52]. Studies have demonstrated that PDL1 + T cells have various tolerogenic effects on tumor immunity through different mechanisms, including induced STAT3-dependent ‘back-signaling’ in CD4 + T cells, inhibition of effector T cells via the PDL1-PD1 axis, and engagement with PD1 + macrophages [53]. In this study, PDL1 was expressed at a low level on PE8T CAR T cells (**sFig.7**), which may be attributed to the longer nonsignaling CAR domain leading to lower activation of PE8T CAR T cells. The presence of endogenous PDL1 in T cells adds complexity to the effects of reverse PD1 signaling in CAR-T cells. This aspect has been generally overlooked in preclinical studies involving CAR T cells and reverse PD1 signaling, including this study, and warrants further investigation in future research.

## Conclusion

In conclusion, the study demonstrates that CAR grafted with PD1 exhibits enhanced antitumor activity with lower cytokine release and no PD1-related off-target toxicity in tumor models that overexpress CD19 and PDL1. Inserting natural immunosuppressive receptors into the CAR structure presents a feasible therapeutic strategy. However, the efficacy and safety of this design should be validated in future clinical trials.

## Electronic supplementary material

Below is the link to the electronic supplementary material.

Supplementary Material 1

## Data Availability

All data generated and analyzed for this study are included in the article/Supplementary Material.

## List of abbreviations

ALL: Acute Lymphoblastic Leukemia
CAR-T: Chimeric Antigen Receptor T-cell
CRS: Cytokine Release Syndrome
EC50: 50% Maximal Effective Concentration
ECAR: Extracellular Acidification Rate
ELISA: Enzyme-Linked Immunosorbent Assay
EM: effector memory
EMD: Extramembrane Domain
FCS: Fetal Calf Serum
GSEA: Gene Set Enrichment Analysis
HD: Hinge Domain
irAEs: Immune-Related Adverse Events
OCR: Oxygen Consumption Rate
PD1: Programmed Cell Death Protein 1
PDL1: Programmed Cell Death Ligand 1
scFv: Antibody Single-Chain Variable Fragment
TCM: Central Memory T Cell
TMD: Transmembrane Domain
TSCM: Stem Cell Memory T Cell
