# Correction: Targeting and cytotoxicity of chimeric antigen receptor T cells grafted with PD1 extramembrane domain

**DOI:** 10.1186/s40164-025-00645-4

**Published:** 2025-04-02

**Authors:** Ang Zhang, Shenyu Wang, Yao Sun, Yikun Zhang, Long Zhao, Yang Yang, Yijian Zhang, Lei Xu, Yangyang Lei, Jie Du, Hu Chen, Lian Duan, Mingyi He, Lintao Shi, Lei Liu, Quanjun Wang, Liangding Hu, Bin Zhang

**Affiliations:** 1 Academy of Military Medical Sciences, Academy of Military Sciences, Beijing, 100850 People’s Republic of China; 2 Senior Department of Hematology, The Fifth Medical Center of Chinese PLA General Hospital, Beijing, 100071 People’s Republic of China; 3 Department of Hematology, Strategic Support Force Medical Center, Beijing, People’s Republic of China; 4 SAFE Pharmaceutical Research Institute Co., Ltd, Beijing, People’s Republic of China

**Correction: Experimental Hematology & Oncology (2023) 12:85** 10.1186/s40164-023-00438-7

In this article [1], Fig. 5B appeared incorrectly and have now been corrected in the original publication.

Image Duplication in Fig. 5B (Mock-T Group on Day 21): There is a duplication of images between the third and fourth mouse. Upon review, we identified that this was due to an inadvertent error during the data archiving process, where the image of the third mouse was mistakenly pasted twice, leading to repetition of both the third and fourth mouse images. The in vivo imaging system number for the third mouse is CLS20191206100438A, and for the fourth mouse, it is CLS20191206101417A.

For readability, the authors optimized the time of the abscissas in Fig. 5C and D in accordance with Fig. 5A.

For completeness and transparency, the old incorrect and correct versions are displayed below.

Incorrect Figure
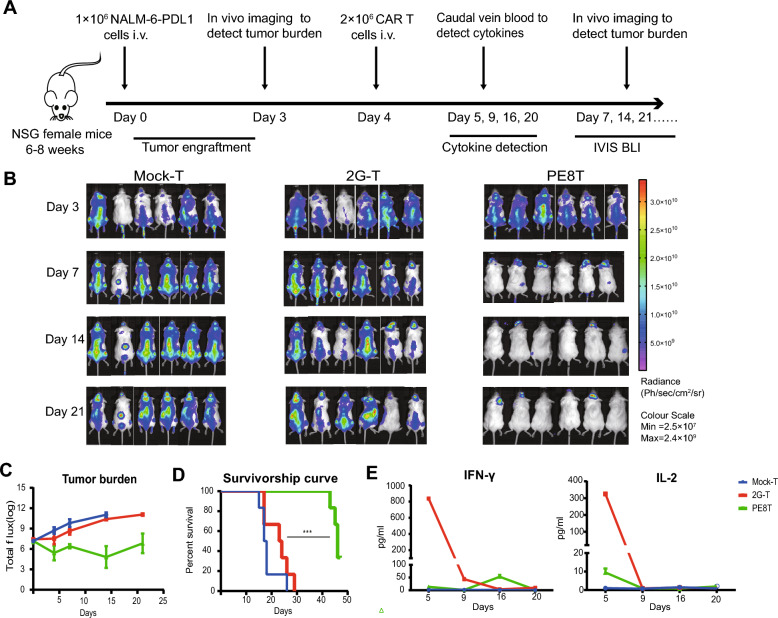


Revised Figure

**Fig. 5:**
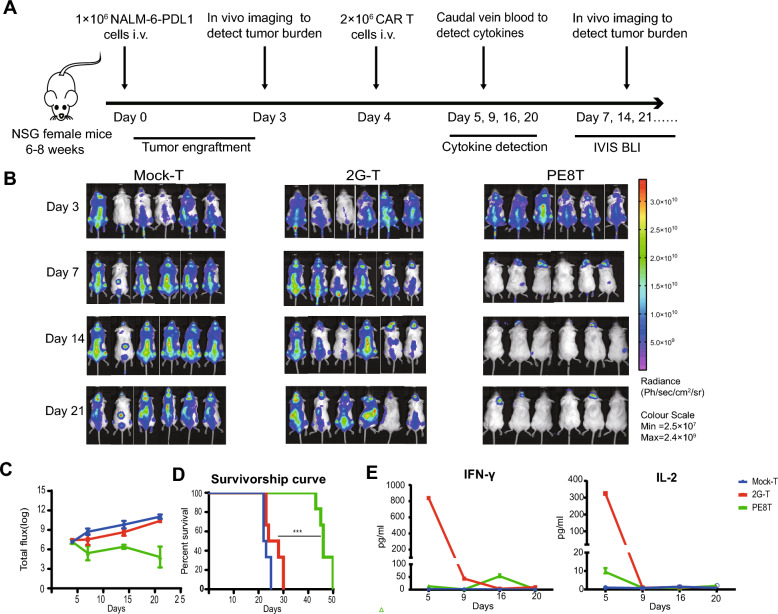
PE8T CAR-T cells harnessed tumor and prolonged survival in vivo compared with 2G CAR-T cells. **A** Schematic representation of the experimental procedure for tumor challenge, T cell adoptive transfer, cytokines detection, and in vivo imaging. **B** Representative bioluminescent images of tumor over time. **C** The logarithm of total body flux (photons/s) for each mouse was quantified and averaged per group (mean ± SEM, n = 6). **D** Kaplan–Meier survival analysis of NALM-6-PDL1-GFP-luc challenged mice. Overall survival curves were plotted using the Kaplan–Meier method and compared utilizing the log-rank (Mantel–Cox) test (n = 6). *P < 0.05; **P < 0.01; ***P < 0.005. **E** On day 5, 9, 16, 20, blood was collected from one group mixed to detect the concentration of IFN-γ and IL-2 using an ELISA-kit (mean ± SD, n = 2)

The original article has been corrected.
