# Supplementary material for: Targeting and cytotoxicity of chimeric antigen receptor T cells grafted with PD1 extramembrane domain
Source: Exp Hematol Oncol. 2023 Sep 30;12:85. doi: 10.1186/s40164-023-00438-7 (PMC10543853; doi:10.1186/s40164-023-00438-7)
Supplement: Supplementary file 1 — Supplementary Material 1 [file 40164_2023_438_MOESM1_ESM.docx]

**SUPPLEMENTAL MATERIAL**

**Targeting and cytotoxicity of CAR-T grafted with PD-1 extramembrane domain**

Ang Zhang^1,3†^, Shenyu Wang^1, 2†^, Yao Sun^2†^, Yikun Zhang^3†^,Long Zhao^2^, Yang Yang^2^, Lei Xu^2^ , Yangyang Lei^2^, Jie Du^4^, Hu Chen^1‡^,^，^Lian Duan^1^, Mingyi He^3^, Lintao Shi^3^, Lei Liu^3^, Quanjun Wang^4^*, Liangding Hu^2^*, Bin Zhang^2^*

1 Academy of Military Medical Sciences, Academy of Military Sciences, Beijing 100850; PR China.

2 Senior Department of Hematology, the Fifth Medical Center of Chinese PLA General Hospital, Beijing 100071; PR China.

3 Department of Hematology, Strategic Support Force Medical Center, Beijing, China

4 SAFE Pharmaceutical Research Institute Co., Ltd.

**†**These authors contributed equally to this work.

**‡** Deceased

Correspondence should be addressed to Zhang Bin: Senior Department of Hematology, the Fifth Medical Center of Chinese PLA General Hospital, Beijing 100071; PR China. Tel +86 010 66 947 125 Email zb307ctc@163.com;

Hu Liangding: Senior Department of Hematology, the Fifth Medical Center of Chinese PLA General Hospital, Beijing 100071; PR China. Tel +86 010 66 947 171 Email huliangding@sohu.com;

Wang Quanjun: SAFE Pharmaceutical Research Institute Co., Ltd, chief scientist, China Tel: +86 18611782967 Email wangquanjunbeijing@163.com


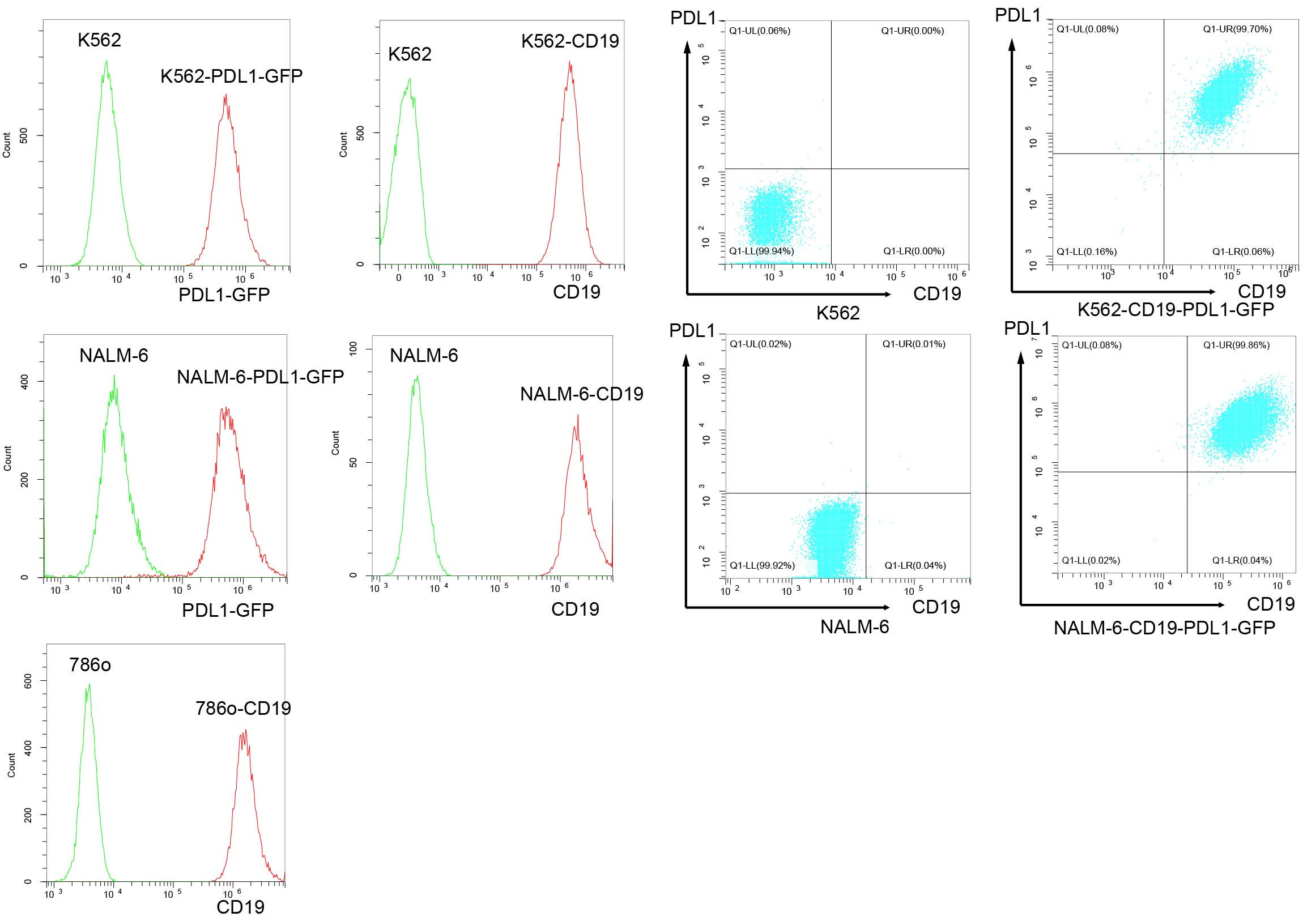
**Figure S1 Characterization of different tumor cells.**

The expression of CD19 and PDL1 on engineered tumor cells was determined by flow cytometry. The GFP protein were co-expressed with PDL1 as an indicator of protein level. GFP, green fluorescent protein.


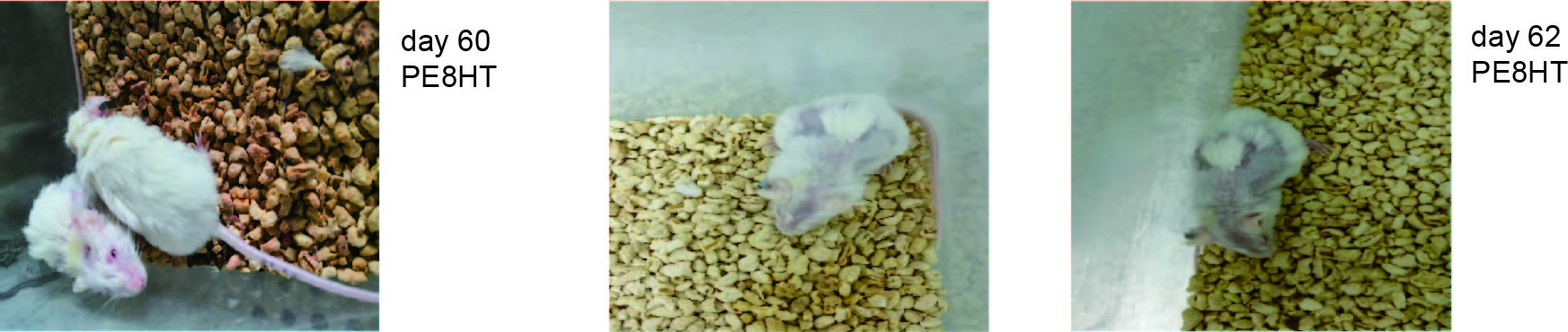


**Figure S2** the mice administered PE8HT CAR-T cells presented severe side effects at day 60 and 62, including depilation and irritability.


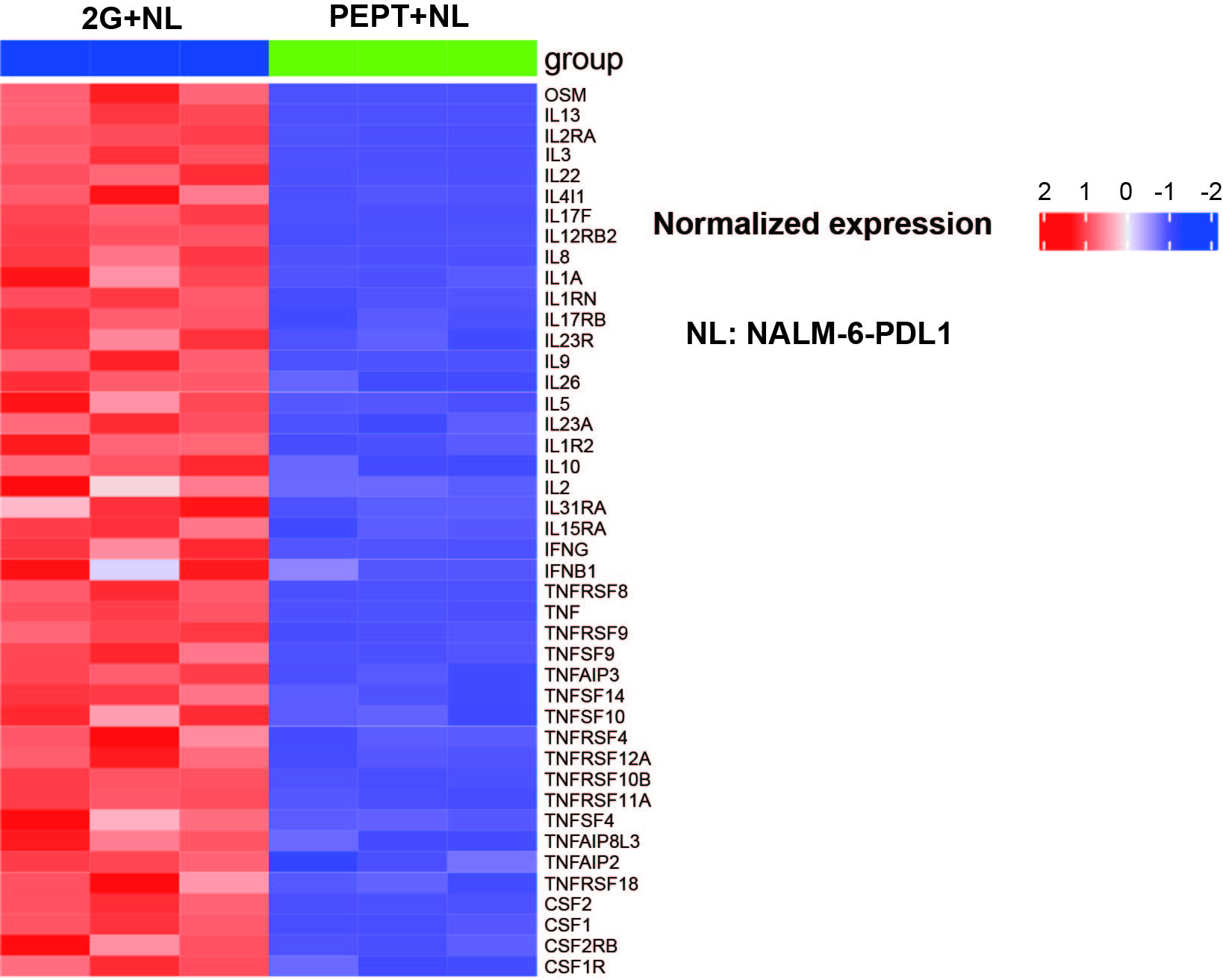


**Figure S3 Heat map of selected cytokines enriched in genes significantly upregulated or downregulated in 2G T vs. PEPT CAR-T cells after stimulation with NALM-6-PDL1 cells at E:T of 1:1 for 48 hours.**

Gene set enrichment analysis (GSEA) revealed that PEPT CAR-T cells exhibited a decrease transcriptome level of proinflammatory cytokines compared to 2G CAR-T cells when stimulated with NALM-6-PDL1 cells. For each pathway, a single sample enrichment score was calculated, and the mean was taken per response group. A color gradient ranging from dark blue to dark red indicates the mean normalized enrichment score (ranging from –2 to +2) of pathways enriched in induced (red) or repressed (blue) genes. The result is mean values ± SD of triplicate from a single experiment.

**
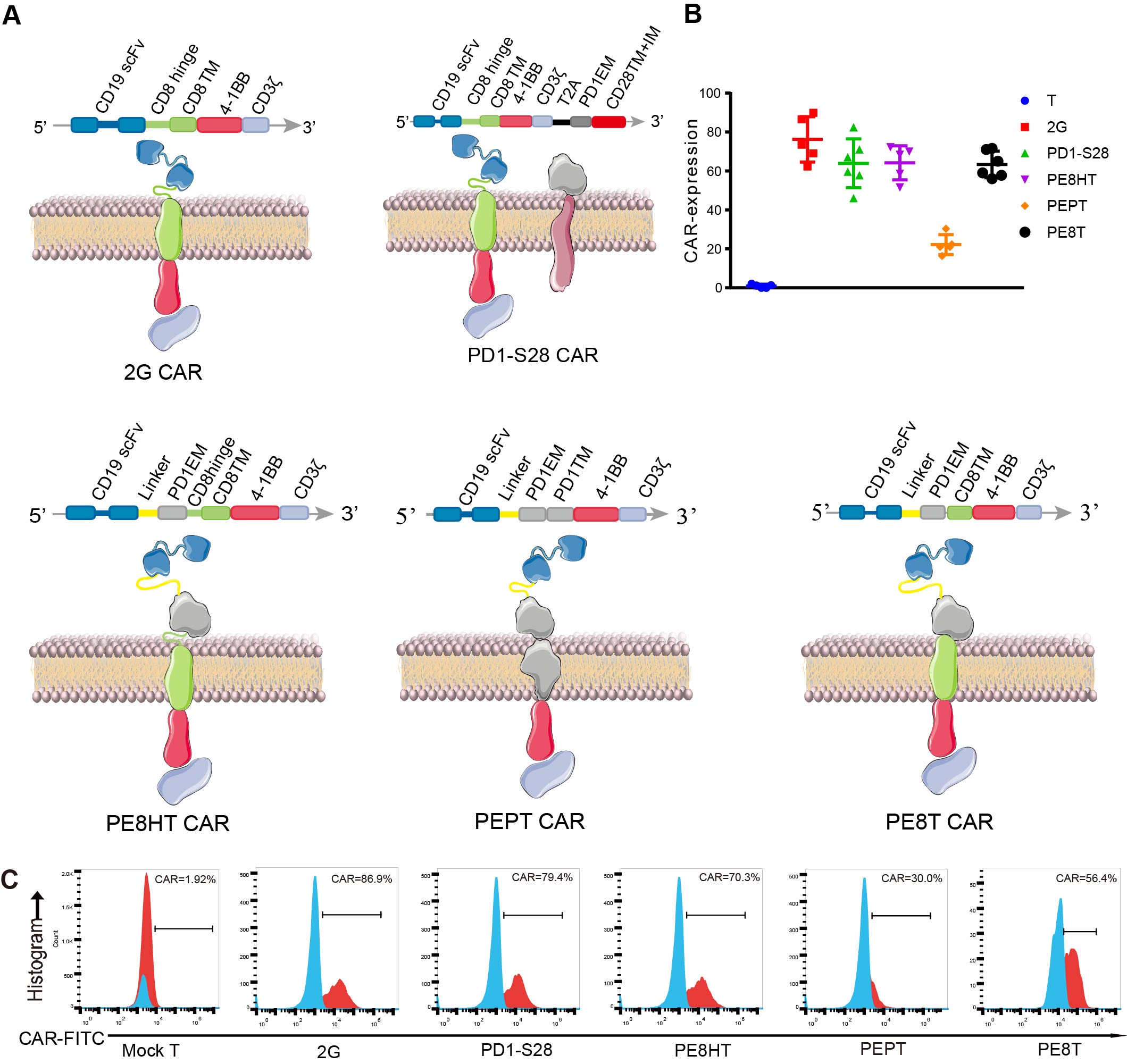
**

**Figure S4 Schematic diagram and expression efficiency of 2G, PD1-S28, PE8HT, PEPT and PE8T**

**A** Diagrammatic model of 2G, PD1-S28, PE8HT, PEPT and PE8T Schematic of five chimeric antigen receptors (CARs) containing variations in the hinge, extramembrane, and transmembrane domains.

1. **C** Expression efficiency of 2G, PD1-S28, PE8HT, PEPT and PE8T on T cells 5-6 days after culture *in vitro* determined by flow cytometry (mean ± SD, n = 5). **B** Results are representative of five experiments with T cells from at least three different healthy donors. **C** Representative plots are shown.


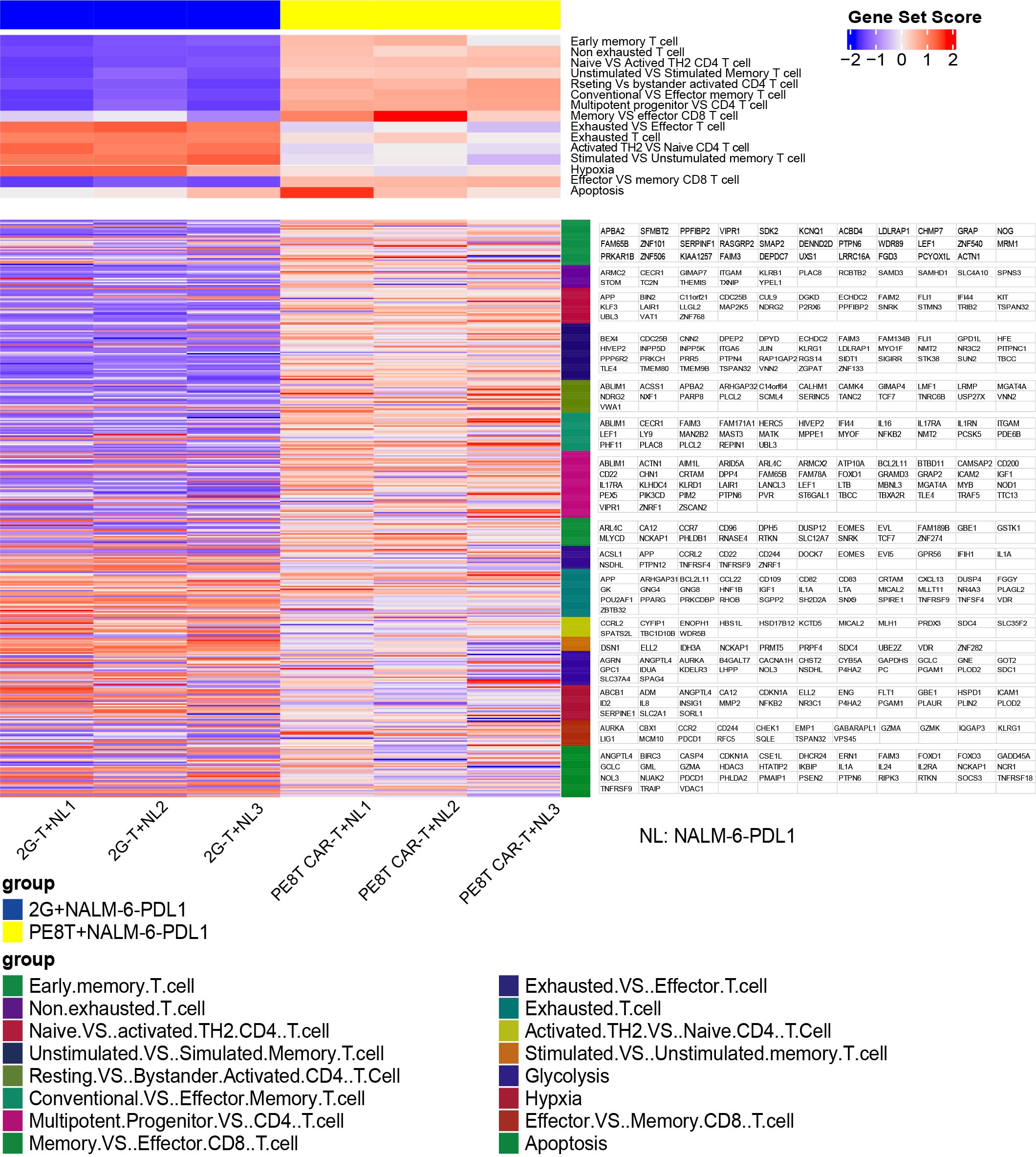


**Figure S5 Heat map of selected pathways enriched in genes significantly upregulated or downregulated in 2G T vs. PE8T CAR-T cells after stimulation with NALM-6-PDL1 cells at E: T of 1:1 for 48 hours.**

PE8T CAR-T cells exhibited enrichment in memory-related genes and demonstrated lower levels of effector differentiation, exhaustion, and apoptosis compared to 2G CAR-T cells when challenged with CD19+PDL1+ tumor cells. For each pathway, a single sample enrichment score was calculated, and the mean was taken per response group. A color gradient ranging from dark blue to dark red indicates the mean normalized enrichment score (ranging from –2 to +2) of pathways enriched in induced (red) or repressed (blue) genes.

**
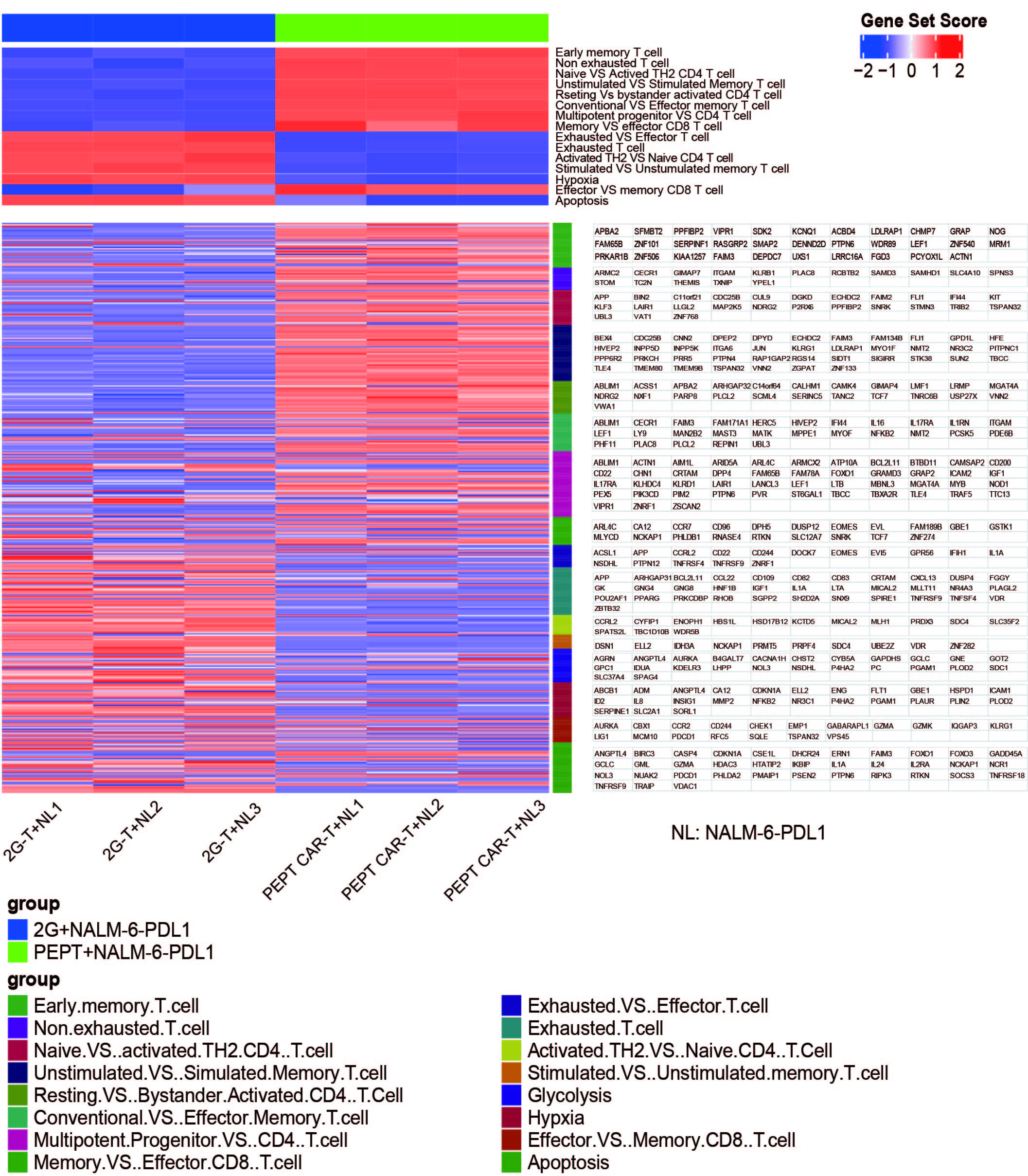
**

**Figure S6 Heat map of selected pathways enriched in genes significantly upregulated or downregulated in 2G T vs. PEPT CAR-T cells after stimulation with NALM-6-PDL1 cells at E: T of 1:1 for 48 hours.**

PEPT CAR-T cells exhibited enrichment in memory-related genes and demonstrated lower levels of effector differentiation, exhaustion, and apoptosis compared to 2G CAR-T cells when challenged with CD19+PDL1+ tumor cells. For each pathway, a single sample enrichment score was calculated, and the mean was taken per response group. A color gradient ranging from dark blue to dark red indicates the mean normalized enrichment score (ranging from –2 to +2) of pathways enriched in induced (red) or repressed (blue) genes.


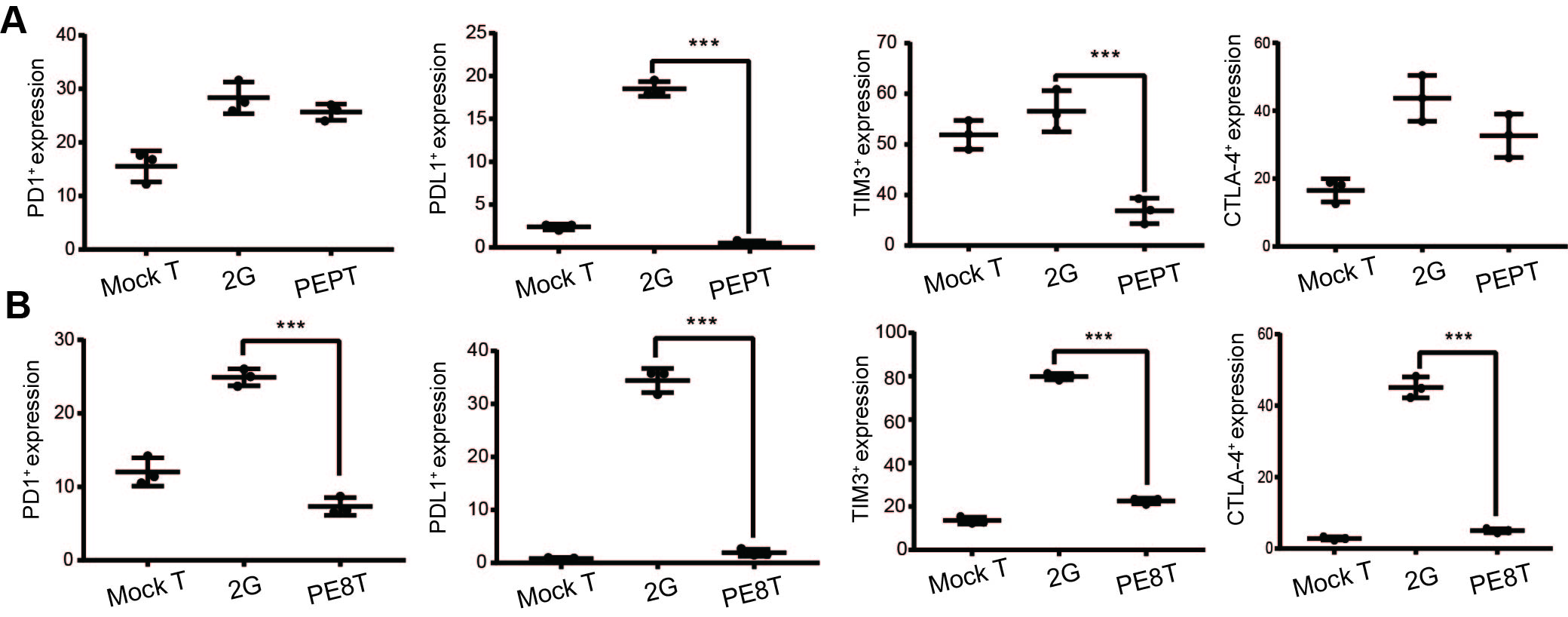


**Figure S7 PEPT and PE8T CAR-T cells incubated with tumor cells exhibit a phenotype of less differentiation and exhaustion by flow cytometry**

The expression of inhibitor receptors, including PD-1, PD-L1, LAG-3, and CTLA-4 on different CAR T cells was measured by flow cytometry after incubation with NALM-6 cells at E:T of 1:1 for 24 h (mean ± SD, n = 3). Data are depicted from three independent experiments with T cells from three healthy donors. **A**, 2G T vs. PEPT CAR-T, **B,** 2G T vs. PE8T CAR-T.
